# The Late Session of the Baltimore College of Dental Surgery

**Published:** 1881-03

**Authors:** 


*The Session of the Baltimore College of Dental Surgery* just terminated, marks an era in this, relatively speaking, venerable institution. The time was in the not remote past when the friends of the old College felt a shade of sadness at the slim attendance on the lectures as contrasted with newer and more prosperous schools further North; and the shade deepened into gloom as the fact was recognized that the South, from which most of the students came, was growing poorer year by year. But that is all past, and with the return of prosperity to the southern community came students flocking from not only the South, but from all quarters; and the class of the past session rolls up almost one hundred names, and fifty-three graduates! Those who have taught these students testify that their average ability is quite equal, if not superior to that of any class in the past. We feel that a larger proportion of really good operators have gone out from the classes of the past few years than ever before; and that more men who will contend for a high professional standing are now educated than at any time in the past. One gratifying fact presents itself,—most of the young men who come to us are better educated in such instruction as will help them in their studies at the college than in previous years. It is to be hoped that in the future no dentist will encourage a student to attend a dental college before he is as well trained mentally as though he expected to enter a law office, or a literary institution for an academic degree. Let this be brought about and a great revolution will have been made in dental education. We have said so often in these pages, that one of the most embarrassing obstacles in the way of teaching a specialty is lack of general training, that it may seem a trite remark. Yet we repeat it. Let the young men be liberally trained, at least so far as good common schooling is concerned, and the professorial labors will be enormously lightened. There are men who enter dental colleges and coolly announce their intention to graduate on their manual dexterity, cleverness of manipulation, &c. Let it be understood that mental training is made a large part of the course of instruction because it is in this that our students are largely wanting, and surely those who prepare them for us will see to it that they are informed in this direction. H.

